# An evaluation of the National Institutes of Health grants portfolio: identifying opportunities and challenges for multi-omics research that leverage metabolomics data

**DOI:** 10.1007/s11306-022-01878-8

**Published:** 2022-04-30

**Authors:** Catherine T. Yu, Brittany N. Chao, Rolando Barajas, Majda Haznadar, Padma Maruvada, Holly L. Nicastro, Sharon A. Ross, Mukesh Verma, Scott Rogers, Krista A. Zanetti

**Affiliations:** 1 Division of Cancer Control and Population Sciences, National Cancer Institute, Rockville, MD USA; 2 Office of Workforce Planning and Development, National Cancer Institute, Rockville, MD USA; 3 Laboratory of Human Carcinogenesis, Center for Cancer Research, National Cancer Institute, Rockville, MD USA; 4 Division of Digestive Diseases and Nutrition, National Institute of Diabetes and Digestive and Kidney Diseases, Bethesda, MD USA; 5 Office of Nutrition Research, National Institutes of Health, Bethesda, MD USA; 6 Division of Cancer Prevention, National Cancer Institute, Rockville, MD USA

**Keywords:** Metabolomics, Multi-omics, Data integration, NIH funding

## Abstract

**Background:**

Through the systematic large-scale profiling of metabolites, metabolomics provides a tool for biomarker discovery and improving disease monitoring, diagnosis, prognosis, and treatment response, as well as for delineating disease mechanisms and etiology. As a downstream product of the genome and epigenome, transcriptome, and proteome activity, the metabolome can be considered as being the most proximal correlate to the phenotype. Integration of metabolomics data with other -omics data in multi-omics analyses has the potential to advance understanding of human disease development and treatment.

**Aim of review:**

To understand the current funding and potential research opportunities for when metabolomics is used in human multi-omics studies, we cross-sectionally evaluated National Institutes of Health (NIH)-funded grants to examine the use of metabolomics data when collected with at least one other -omics data type. First, we aimed to determine what types of multi-omics studies included metabolomics data collection. Then, we looked at those multi-omics studies to examine how often grants employed an integrative analysis approach using metabolomics data.

**Key scientific concepts of review:**

We observed that the majority of NIH-funded multi-omics studies that include metabolomics data performed integration, but to a limited extent, with integration primarily incorporating only one other -omics data type. Some opportunities to improve data integration may include increasing confidence in metabolite identification, as well as addressing variability between -omics approach requirements and -omics data incompatibility.

**Supplementary Information:**

The online version contains supplementary material available at 10.1007/s11306-022-01878-8.

## Introduction

The application of high-throughput profiling techniques to interrogate the genome, epigenome, transcriptome, proteome, metabolome, and microbiome has supported detailed molecular examinations into human health and disease (Hasin et al., 2017). While earlier -omics studies focused on a single -omics profile to characterize associations between biological molecules occurring within that profile and a phenotype, more recent advances in profiling techniques and technologies have brought about an expansion into research that leverages integration of multiple -omics data (Cavill et al., 2016). Multi-omics studies aim to combine data generated from different -omics technologies together to gain understanding of how different -omics signatures relate to one another, and the direction of those interactions (Sun & Hu, 2016). They improve our understanding of the basic underlying biology and systems-level and physiological processes by using a comprehensive approach to investigate human health systems (Cavill et al., 2016).

**Fig. 1:**
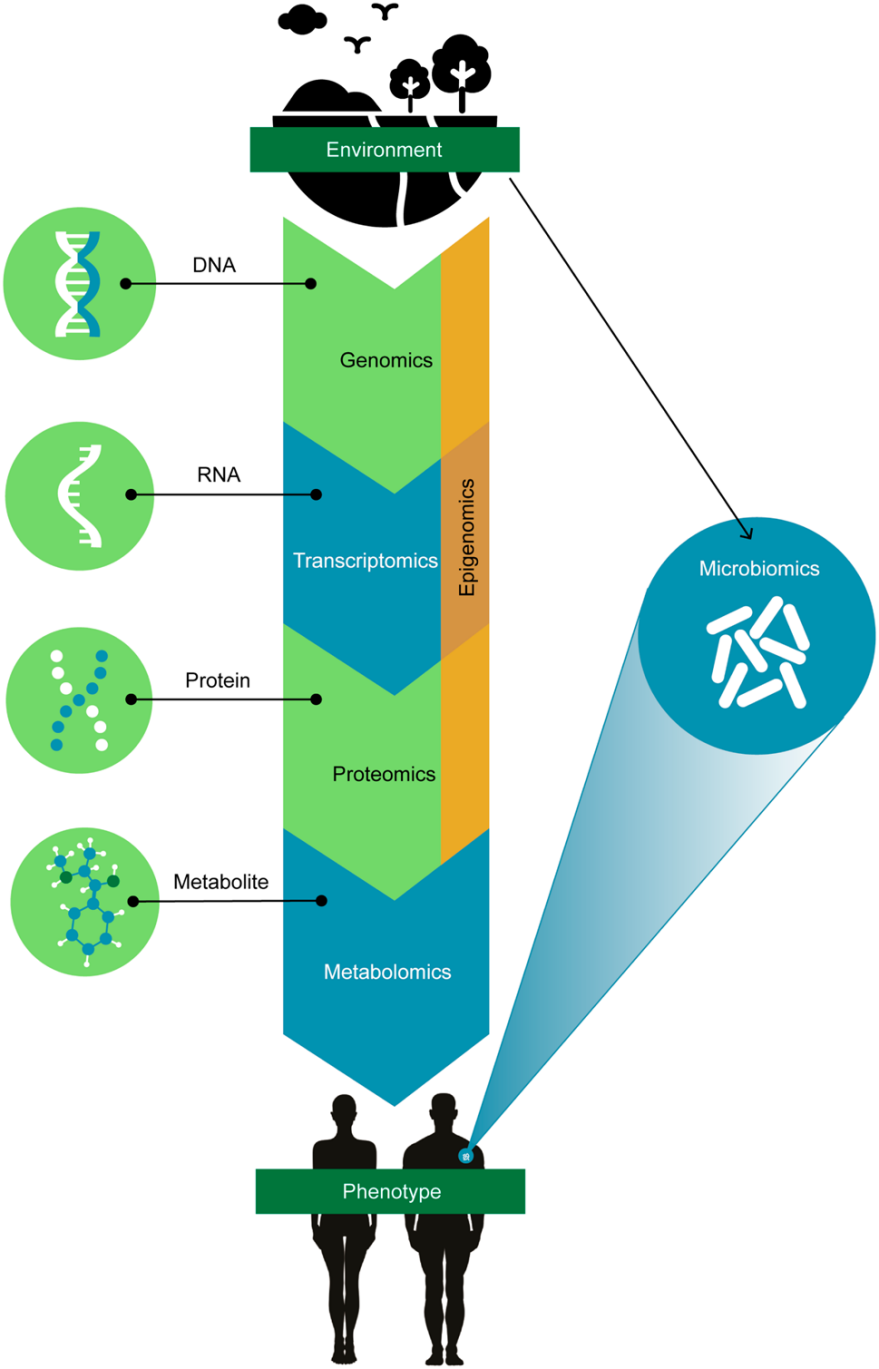
Overview of how various -omics sciences interact with one another, the environment, and phenotype. Epigenomics is portrayed as spanning the several -omics sciences to demonstrate that in addition to environmental exposures, physiological activity occurring at genomic, transcriptomic, and proteomic levels influence epigenomic status. Commensal microorganisms residing within (gut and other organs) and on (skin) the human body, collectively known as the microbiome, are largely influenced by host-microbe interactions that reflect in the microbiomic compositional and functional profiles

One -omics technology with possible utility in multi-omics studies is metabolomics. Metabolomics is the systematic large-scale study of the small molecular weight products of metabolism that provides a tool for biomarker discovery and improving disease monitoring, diagnosis, prognosis, and treatment response, as well as for delineating disease mechanisms and etiology (Liesenfeld et al., 2013; Nicholson & Lindon, 2008; Patti et al., 2012). Systemic metabolite repertoire is affected by both external exposures and physiological activity, which includes exogenous environmental factors and endogenous processes occurring at higher biochemical levels (Fiehn, 2002; Tzoulaki et al., 2014). As a downstream product of the genome and epigenome, transcriptome, and proteome activity, the metabolome can be considered as being the most proximal correlate to the phenotype (Fiehn, 2002). Thus, when included in multi-omics and integrative study designs, metabolomics can complement upstream -omics data to provide highly informative biochemical insights into diseases and other physiological endpoints (Jendoubi, 2021; Worheide et al., 2021). Figure 1 demonstrates how different -omics profiles relate to one another, the exposome, and phenotype.

In order to better understand the applications and potential limitations of human studies employing multi-omic data collection that includes metabolomics, we evaluated National Institutes of Health (NIH)-funded grants for use of metabolomics data with data of another -omics type. The objective of this study was two-fold. First, we aimed to determine what types of multi-omics studies, defined as studies that collect more than one -omics data type, included metabolomics data collection. Then, we looked at those multi-omics studies to examine how often grants employed an integrative analysis approach using metabolomics data. To achieve this objective, we described the funding by type of award, disease phenotype, biospecimen type, and whether other -omics data were integrated with metabolomics data. This analysis is intended to help identify limitations for the inclusion of metabolomics data in multi-omics research, including whether these data were being integrated with other -omics data. Understanding how metabolomics data is currently being leveraged in an integrative multi-omics manner can inform future directions of the field that could lead to improving the diagnosis and prognosis of disease.

## Methods

The authors of this study included NIH-funded grants employing metabolomics in multi-omics analyses that were active on May 8, 2019 in the analysis presented in this manuscript. The authors analyzed the grants portfolio using NIH’s Query View Report (QVR) software. Grants were identified as relevant if they contained one *or* more of the metabolomics terms from the first column in Table 1 *and* one *or* more of the search terms from at least one of the subsequent columns in Table 1. This search resulted in the identification of 562 grants. Grants were then screened to determine if they met the following inclusion criteria: (1) the project uses human biospecimens either newly collected in the study grant or uses previously collected samples, (2) metabolomics analysis is performed on the human biospecimens, and (3) the project performs hypothesis-driven metabolomics research as part of a defined analytical plan. Grants that used existing metabolomics data were included in the analysis. For the purpose of this study, we aimed to be inclusive of a broad range of metabolomics grants and included both untargeted metabolite profiling (global approach) and semi-targeted profiling (up to 400 metabolites) in our definition of metabolomics (Dunn et al., 2011). Targeted analyses examining typically less than 20 metabolites which are related in function or class were not considered metabolomics (Dunn et al., 2011). During the data extraction, we determined that 330 grants fully met the inclusion criteria and were included in the study.

**Table 1:** Grant portfolio search criteria

One *or* more terms from column 1	One *or* more terms from at least one other column 2–6				
Metabolomics	Genomics	Epigenomics	Transcriptomics	Proteomics	Microbiome
Metabolomic(s) Metabolome Metabonomic(s) Metabonome Metabolic profile Metabolite profile Metabolic signature	Genome Genomic(s) Genetic(s) Gene(s) SNP(s) Mutation DNA RNA Variant(s) Loci Locus	Epigenome Epigenomic(s) Epigenetic(s) Methylation CpG island miRNA	Transcriptome Transcriptomic(s) TWAS	Proteome Proteomic(s)	Microbiome Microbiota Microbial Microbe(s) Bacteria Bacterial Virus Viral

The authors of this manuscript worked in pairs to review the abstracts and specific aims of all grants and extracted data including disease phenotype, biospecimen type, -omics data types collected, and whether metabolomics data were integrated with other -omics data types. Grants were denoted as performing integration if they specified that metabolomics data would be analyzed with data from at least one other -omics analysis. Discordance between authors’ answers was flagged using SAS 9.4 following data extraction. We resolved all discordance first between the reviewer pairs and then through a group discussion consisting of all manuscript authors when the reviewer pairs could not reach consensus. Additionally, during this review phase, grants were verified for whether they included multi-omic data collection, which was defined as collecting at least one additional data type (genomics, epigenomics, transcriptomics, proteomics, or microbiomics). Grants that collected metabolomics data only were excluded from the analysis. Through the data extraction, we determined that 197 grants met our inclusion criteria of performing multi-omics analyses that include metabolomics data collection and 123 of these grants used an integrative analysis approach.

**Fig. 2:**
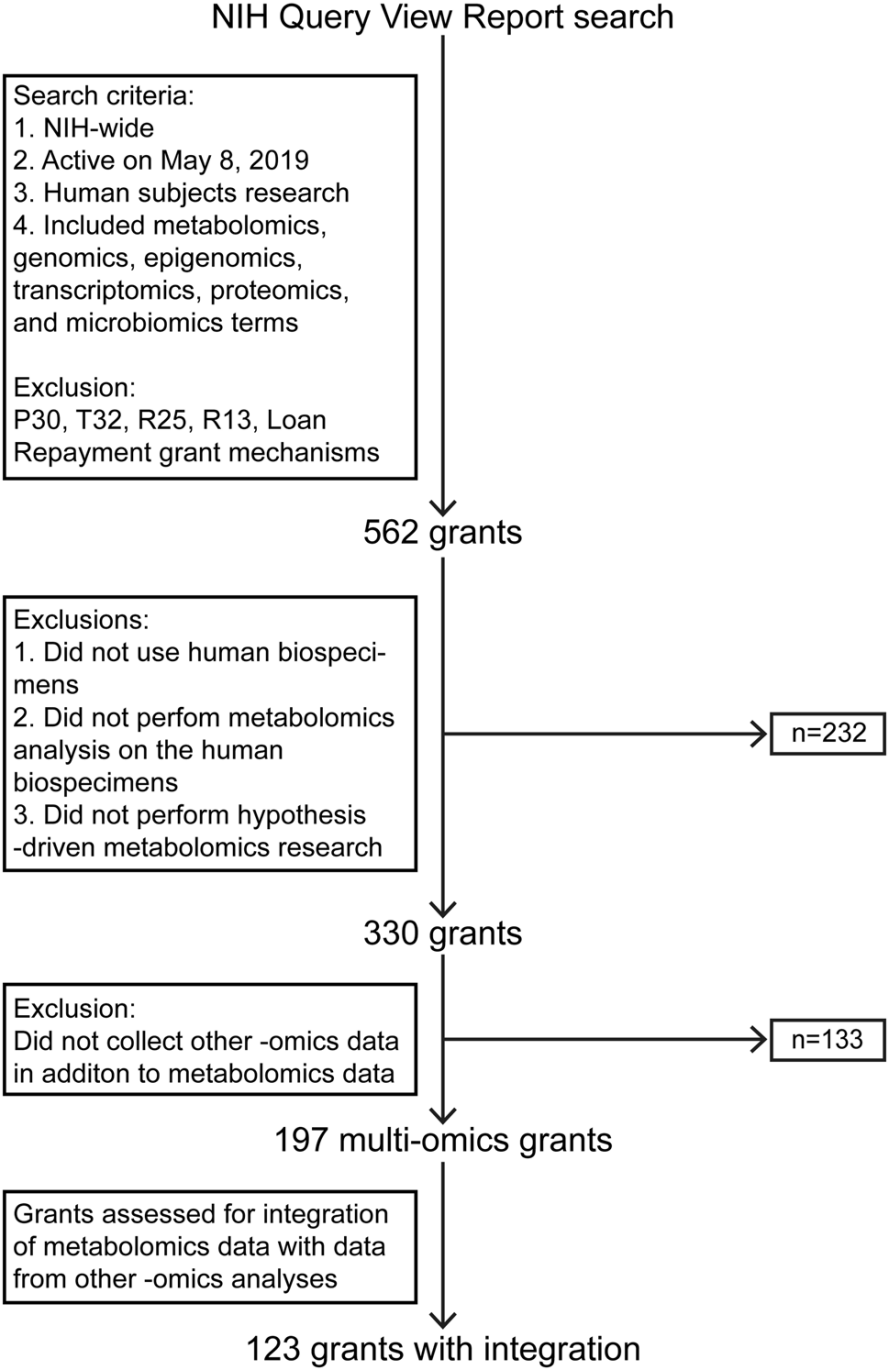
A summary of the methods followed for generating the analyzed portfolio of NIH-funded grants

**Fig. 3:**
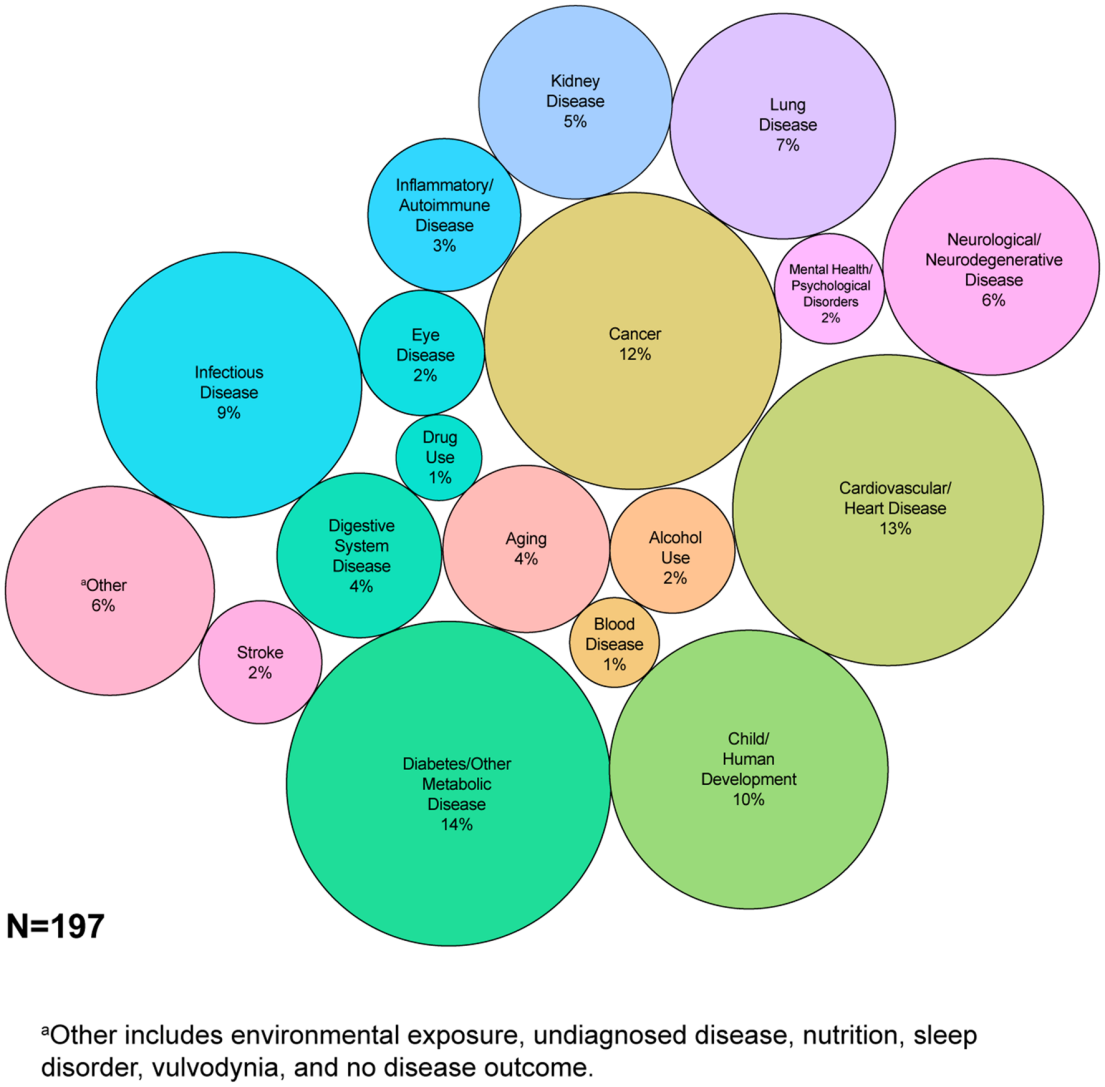
Circle packing chart displays the percentage of NIH-funded grants employing metabolomics in multi-omics studies in the disease state studied

**Fig. 4:**
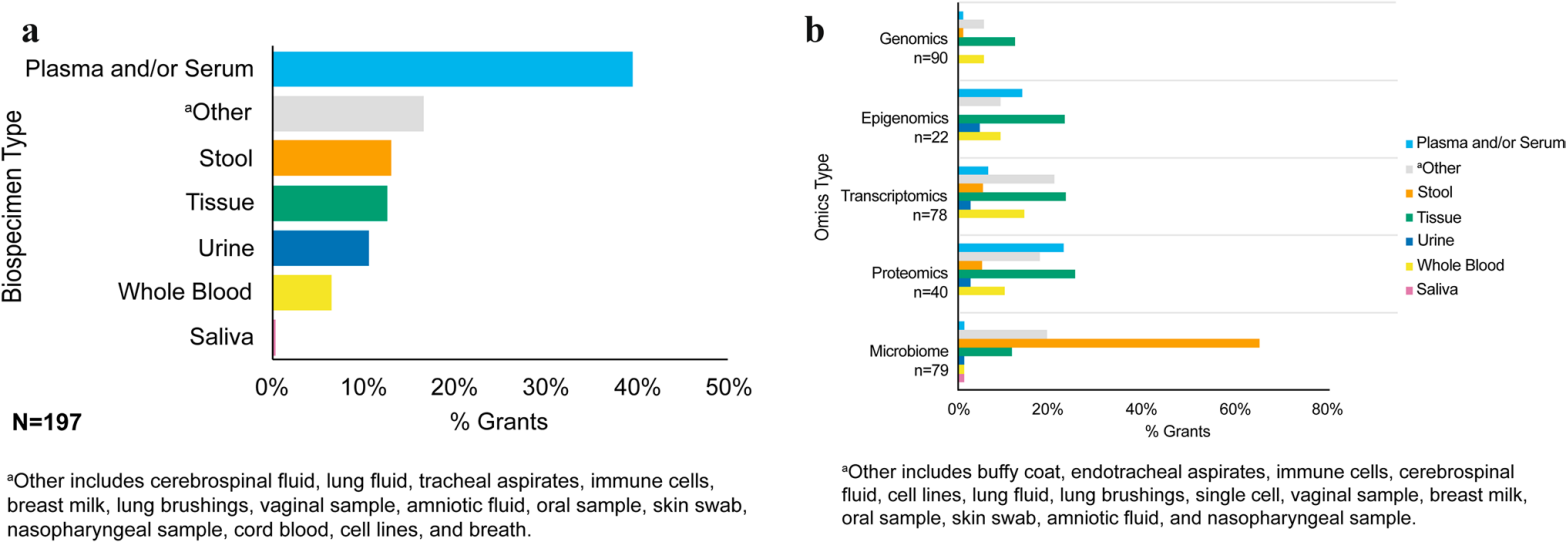
**a** Bar graph displays commonly used biospecimen types for metabolomics analyses that were specified in the abstract and specific aims of NIH-funded grants employing metabolomics in multi-omics studies. Some grants are represented by multiple data points because a single study may have used more than one biospecimen type for metabolomics analyses. **b** Bar graph shows the breakdown of biospecimen types used for genomics, epigenomics, transcriptomics, proteomics, and microbiomics analyses that were specified in the abstract and specific aims of NIH-funded grants employing metabolomics in multi-omics studies. Some grants are represented by multiple data points because a single study may have used more than one biospecimen type for each -omics technology

**Fig. 5:**
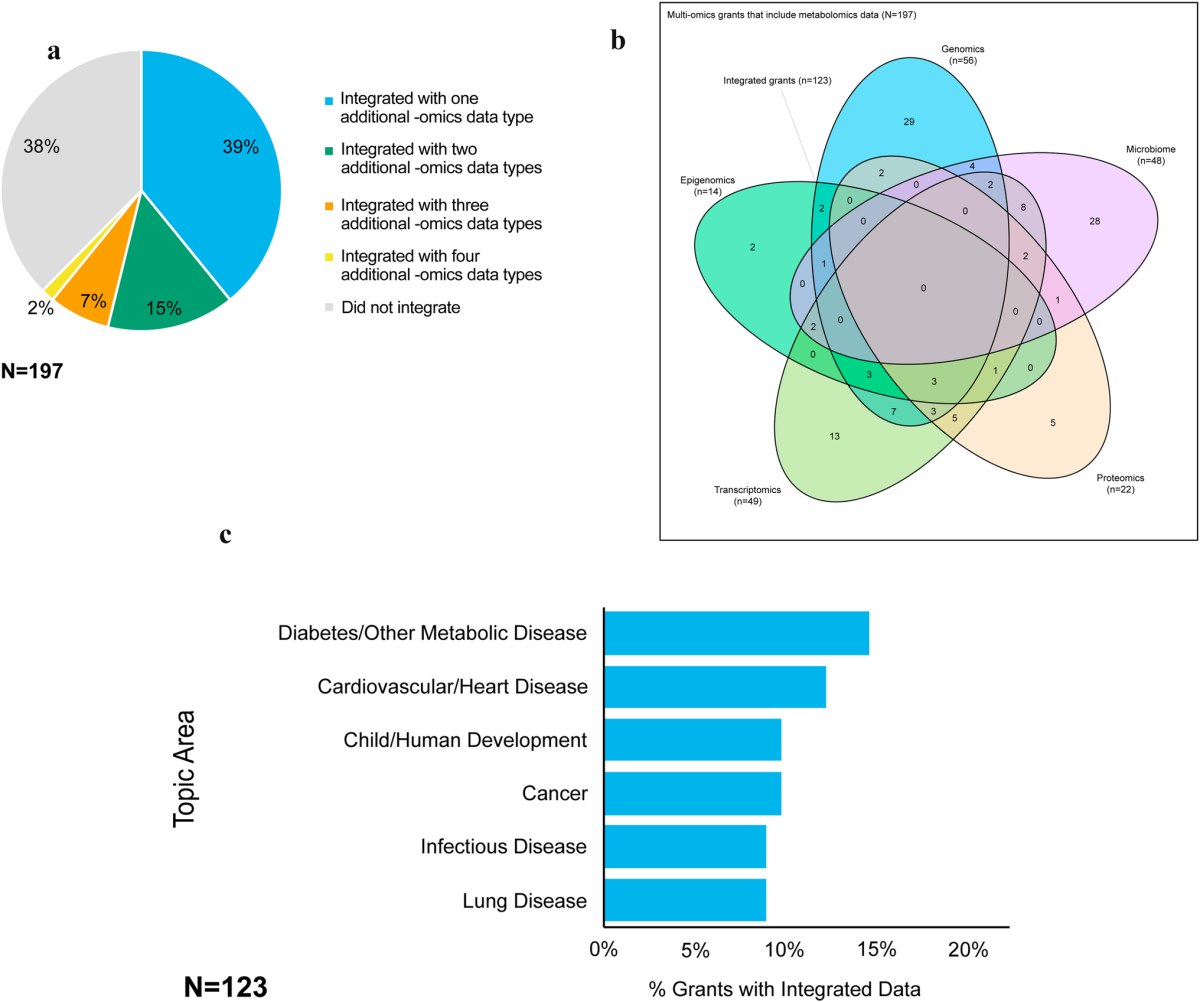
**a** Pie chart depicts the percentage of NIH-funded grants in which metabolomics data were integrated with other -omics data in multi-omics studies, including genomics, epigenomics, transcriptomics, proteomics, and/or microbiomics data. No grants integrated all six -omics data types in the multi-omics set. **b** Venn diagram displays the number of NIH-funded grants employing metabolomics in multi-omics and integrative analysis approaches where metabolomics data were integrated with -omics data from one or more additional -omics technologies, including genomics, epigenomics, transcriptomics, proteomics, and/or microbiomics. **c** Bar graph depicts the percentage of NIH-funded grants employing metabolomics in multi-omics studies where metabolomics data were integrated with one or more other -omics data in the top four diseases studied

A summary of the methods followed for generating the analyzed portfolio is presented in Fig. 2. All analyses were performed using Excel Version 2108. Figures 4a, 4b, 5a, and 5c were created in Excel Version 2108. R Studio Version 1.3.1093 was used to create Fig. 3 and Online Resource 2.

## Results

The grant search strategy identified 562 grants. After screening grants by applying our inclusion and exclusion criteria, 197 of the 562 originally identified grants were included in the analysis. Research Grants (R series) comprised 67% (n = 132) of the grants (data not shown). Cooperative Agreements (U series) (n = 34, 17%) were the next most common grants, followed by Career Development Awards (K series) (n = 19, 10%) (data not shown). A description of award types is listed in Online Resource 1. The majority of NIH-funded multi-omics grants with a metabolomics component focused on diabetes and other metabolic diseases (n = 27, 14%) (Fig. 3). The next most commonly studied disease state was cardiovascular disease (n = 25, 13%), followed by cancer (n = 23, 12%) and child and human development (n = 19, 10%) (Fig. 3).

A variety of biospecimens (biological fluids, solid tissue, or exfoliated cells) were used for different -omics analyses. The type of biospecimen used for analysis varied based on the -omics technology employed (Figs. 4a and 4b). For metabolomics analyses, plasma and serum were primarily used (n = 78, 40%) (Fig. 4a). Tissue was most commonly used in genomics (n = 11, 12%), epigenomics (n = 5, 23%), and transcriptomics analyses (n = 18, 23%) (Fig. 4b). For proteomics analyses, tissue was primarily used (n = 10, 25%), followed by plasma and serum biospecimens (n = 9, 23%) (Fig. 4b). Stool samples were the most common biospecimen used in grants performing microbiomics analyses (n = 51, 65%) (Fig. 4b).

The majority of grants that collected multi-omics data used an integrative analysis approach that included metabolomics data (n = 123, 62%). There were 77 (39%) grants that integrated metabolomics data with data from one other -omics approach, 29 (15%) grants that integrated metabolomics data with data from two additional -omics approaches, 14 (7%) grants that integrated metabolomics data with data from three additional -omics approaches, and 3 (2%) grants that integrated metabolomics data with data from four additional -omics approaches (Fig. 5a). No grants in the examined portfolio integrated data across all six of the -omics approaches investigated in this paper: genomics, epigenomics, transcriptomics, proteomics, metabolomics, and microbiomics.

Figure 5b shows a Venn diagram detailing the -omics combinations in the 123 NIH-funded multi-omics grants integrating data with metabolomics data. The majority of grants that integrated -omics data combined metabolomics data with genomics data (n = 56, 46%). The next most frequent -omics data that were integrated with metabolomics data were transcriptomics data (n = 49, 40%), followed by microbiomics data (n = 48, 39%). Metabolomics data were integrated with epigenomics data at the lowest rate (n = 14, 11%). Of the 77 grants that integrated metabolomics data with only one other -omics data type, all possible pairs were represented (i.e., metabolomics and genomics, metabolomics and epigenomics, metabolomics and transcriptomics, metabolomics and proteomics, and metabolomics and microbiomics); most of these grants investigated metabolomics and genomics jointly (n = 29, 24%). For grants integrating metabolomics data with two other -omics data types, there were seven combinations represented: metabolomics with genomics and epigenomics (n = 2, 2%), genomics and transcriptomics (n = 7, 6%), genomics and proteomics (n = 2, 2%), genomics and microbiomics (n = 4, 3%), transcriptomics and proteomics (n = 5, 4%), transcriptomics and microbiomics (n = 8, 7%), and proteomics and microbiomics (n = 1, 1%). For grants integrating metabolomics data with three other -omics data types, there were also seven combinations represented. The combinations most represented in this category were the integration of metabolomics data with genomics, epigenomics, and transcriptomics data (n = 3, 2%), as well as the integration of metabolomics data with genomics, transcriptomics, and proteomics data (n = 3, 2%). Three grants (2%) integrated metabolomics data with genomics, epigenomics, transcriptomics, and proteomics data jointly. This was the maximum combination represented where metabolomics data were integrated with four other -omics data types.

For the 123 grants that used an integrative analysis approach that included metabolomics data, most grants were Research Grants (n = 84, 68%), followed by Cooperative Agreements (n = 20, 16%) and then Career Development Awards (n = 12, 10%) (data not shown). The majority of grants that integrated -omics data focused on diabetes and other metabolic diseases (n = 18, 15%) (Fig. 5c). Cardiovascular disease was the next most common disease outcome represented in grants that integrated -omics data (n = 15, 12%), followed by child and human development (n = 12, 10%) and cancer (n = 12, 10%) as the third most common, and infectious disease (n = 11, 9%) and lung disease (n = 11, 9%) as the fourth most common (Fig. 5c). Additional data on the full breakdown of topic areas studied by grants that integrated -omics data are given in Online Resource 2.

The data used to conduct this portfolio analysis can be found in Online Resource 3.

## Discussion

The metabolome reflects the downstream products of multiple interactions between genes, transcripts, proteins, and metabolites (Fig. 1); therefore, we included in this analysis genomics, transcriptomics, and proteomics. We also included epigenomics, which captures the transgenerational and environmental impact on the genome, and microbiomics to capture microbial composition and function and their metabolites to comprehensively examine the types of -omics data that are being integrated with metabolomics data to study disease in humans. Pinu et al. (2019) explained that the analytical and data integration components that are critical to metabolomics studies are compatible with genomics, transcriptomics, and proteomics studies. The metabolome being in close proximity to cellular or tissue phenotypes provides what the authors term a ‘common denominator’ to multi-omics study design, including guidelines for sample collection, handling and processing, and experimental analyses (Pinu et al., 2019). In turn, knowledge of metabolomics by researchers focusing on other -omics or multi-omics studies may offer the opportunity to improve studies using an integrated -omics approach. Therefore, understanding the characteristics of multi-omics studies that include metabolomics data analyses will allow a better understanding of the current state of the field.

The majority proportion of grants were Research Grants, representing 67% of multi-omics grants in the examined portfolio. Though Career Development Awards represented the third most common grant program type, they represented only 10% of grants. Increased funding to support early career investigators could provide a mechanism for trainees to master the technical and analytical skills required for conducting multi-omics approaches. However, it may not be feasible to expect early career investigators to become experts in all of the complex technical and analytical skills used across the multiple -omics sciences. The conduct of these studies is likely better suited for grants with budgets that can accommodate the various expertise needed to manage the diverse sampling requirements, analytical platforms, pre-processing methods, data structure, and data analysis methods required in multi-omics studies.

Of the 123 grants that used an integrative analysis approach, genomics data were integrated more than any other type of -omics data in these grants. Genomics can identify genetic variants associated with disease, but integration of genomics data with metabolomics data can help narrow the causal mechanisms of the identified variants by explicating the functionality of disease-associated genetic variants (Karczewski & Snyder, 2018). The next most common -omics data that were integrated with metabolomics data were transcriptomics data, followed by microbiomics data. Transcriptomics can cross-sectionally measure levels of gene expression in a cell or organism and does so at an intermediate level from phenotype (Karczewski & Snyder, 2018). A combined analysis of metabolomics data with transcriptomics data from the same sources may reveal functional mechanisms that link genes to phenotype (Chu et al., 2019). Microbiomics allows characterization of microbiome compositional profiles that may be associated with disease (Hasin et al., 2017). However, a limitation of microbiome profiling is the difficulty in discerning precisely how the microbiome is directionally related to a phenotype (Karczewski & Snyder, 2018). Furthermore, microbiome data collected to date usually covers only bacteria and does not include fungi, archaea, and bacteriophages, which comprise the whole microbiota. Metabolomics can complement microbiomics to help tease out time relationships between the two disciplines, especially when longitudinal sampling is practiced and short measurement intervals are used (Chu et al., 2019). Increased integration of metabolomics data with other -omics data presents an opportunity for identifying causal and functional interpretations of disease.

The majority of multi-omics grants in the examined portfolio integrated metabolomics data with other -omics data, but to a limited degree. When grants integrated metabolomics data with other -omics data, integration was more likely to be with only one other -omics approach. This result suggests that there are challenges with integrating various layers of -omics data in multi-omics analyses or that the original study design may not support multi-omics research questions beyond integration of two types of -omics data. To broaden integration to include more -omics data types, various barriers need to be overcome. One challenge complicating successful data integration across -omics disciplines is that -omics data are difficult to reproduce and compare because -omics data are noisy, heterogeneous, and largely qualitative (Pinu et al., 2019; Subramanian et al., 2020). For example, batch effects of the sample runs contribute to -omics data heterogeneity and can confound integration of multi-omics data. Researchers should select a data integration strategy (e.g., composite network approach, simultaneous integration) that best addresses heterogeneity between multi-omics datasets in their study (Worheide et al., 2021). Furthermore, inherent challenges with metabolomics data may limit wider integration of multi-omics data since integrative analyses compound the issues of each -omics data type included in a multi-omics set. At this time, only a small percentage of metabolomics data is decipherable and well-annotated based on publicly available databases (Dunn et al., 2013; Mahieu & Patti, 2017). Although many NIH studies deposit metabolomics data in the National Metabolomics Data Repository (NMDR), formerly Metabolomics Workbench, the data lack the level of standardization that may be required to include metabolomics into multi-omics studies (NIH Common Fund's National Metabolomics Data Repository; Smirnov et al., 2021). Additionally, NMDR does not currently accept controlled data, which limits the deposition of clinical and epidemiological studies. This has resulted in some NIH-funded clinical and epidemiological studies depositing metabolomics data into the controlled-access database of Genotypes and Phenotypes (dbGaP), which was designed to accept genotype and related phenotype data and is not standardized for metabolomics data. However, most data repositories are not designed to hold multi-omics data. Thus, for multi-omics studies using existing data, it is challenging for researchers to access data that reside in different locations or databases and work with multiple datasets in an accessible and convenient manner. It is especially difficult to access sample-level-matched multi-omics datasets, assuming that this is even possible (Tarazona et al., 2021). As for the challenge of unidentified metabolites in metabolomics data, improved compound identification and characterization efforts increase confidence in metabolite identity (Chaleckis et al., 2019; Dunn et al., 2013). Enhanced confidence in metabolite identity and accuracy of metabolomics data will encourage efforts to integrate other -omics data with metabolomics data (Creek et al., 2014; Jendoubi, 2021). The NIH Common Fund Metabolomics Compound Identification Program is currently addressing several of these issues for metabolite chemical and bioinformatic analysis (National Institutes of Health Common Fund). There are several commercial entities such as Metabolon and Nightingale that provide metabolite identification as part of their metabolomics analysis services. Similar complementary and robust efforts are currently underway in the metabolomics research community for compound identification, making further strides in the progress. More broadly, improving the availability and use of reference standards, quality control measures, and access to standardized operating protocols can ensure that data generated from -omics approaches are of high validity and quality (Pinu et al., 2019). Measuring -omics data in a quantitative manner is expected to promote multi-omics data integration since quantitative -omics measurements can obtain greater analytical precision and accuracy compared to non-quantitative measurements, helping to assure study reproducibility and comparability in multi-omics studies (Pinu et al., 2019). While there are a few considerations regarding quantitative metabolomics, such as biological variability, sample preparation, choice of analytical platforms, and data analysis approaches, efforts have been underway to address these important aspects. For example, advancements in the technological approaches, such as ultra-high-resolution mass spectrometry and use of internal standards when possible, have significantly aided in improvements with regards to quantitative metabolite measurements (Perez de Souza et al., 2021). Multi-omics data integration can further be facilitated through collection of sufficient meta-data on samples, information essential for making biologically pertinent and contextually correct interpretations of results (Pinu et al., 2019). Furthermore, there is a need for improved open-source pathway databases and updated network models for the generation of clear connections between multi-omics data levels (Eicher et al., 2020). Additionally, another limitation of multi-omics analysis is that it is resource intensive to integrate data, such that the relative effort and resources required for data integration greatly exceeds that of data generation with each additional -omics type included in a multi-omics set (Palsson & Zengler, 2010). The need for increased effort and resources can be attributed to the considerable obstacles that must be overcome during the search for meaningful associations in multi-omics studies. Tarazona et al. (2021) explains some of these challenges that limit our ability to integrate high-throughput -omics data, specifically (1) heterogeneity across various technologies used to collect -omics data, (2) imputation of missing values, (3) challenges related to interpretation of multilayered systems models, and (4) issues associated with data annotation and storage and computational resources. Furthermore, study design considerations are imperative for the data integration steps as this has direct implications on the data analysis (Cavill et al., 2016).

Diabetes and other metabolic diseases, cancer, cardiovascular disease, and child and human development being the most studied topic areas was an expected result as the focus of this paper was to identify grants with a known metabolomic focus and each of these outcomes involve changes to cellular metabolism pathways. Diabetes, cancer, and cardiovascular disease are also among the leading causes of death in the United States (Kochanek et al., 2020). As high priority research areas, there are likely to be more resources (cohorts, biospecimens, and -omics data) available to be leveraged for studying these outcomes compared to outcomes with less extensive disease burden. When looking at the subset of grants integrating other -omics data with metabolomics data, diabetes and other metabolic diseases, cardiovascular disease, cancer, and child and human development remained in the top three most commonly studied outcomes. Genomics data were most commonly leveraged alongside metabolomics data in the grants that integrated -omics data and focused on diabetes and cardiovascular disease (data not shown). Transcriptomics data were most often integrated with metabolomics data in the integrated grants studying cancer (data not shown). When the focus was on child and human development, microbiomics data were more often combined with metabolomics data than other -omics data (data not shown).

Further examining those studies that integrated other -omics data with metabolomics data, we observed lung disease and infectious disease emerge as top topic areas studied in these grants. In these studies investigating lung disease, we primarily saw integration of metabolomics data with transcriptomics data and with genomics data (data not shown). Among studies that have examined lung disease using a data integration approach, transcriptomics data being a component has revealed new candidate causal genes for chronic obstructive pulmonary disease, as well as helped provide a more detailed and explanatory image of asthma biology (Kelly et al., 2018; Lamontagne et al., 2018). The integration of genomics data with metabolomics data to study asthma has enabled identification of novel predictors of asthma control (McGeachie et al., 2015). In the integrated grants examining infectious disease, metabolomics data were primarily integrated with transcriptomics data. The application of integrated transcriptomics and metabolomics data analyses to study Epstein-Barr virus infection has revealed selective therapeutic targets for treating lymphoid cancers associated with the virus (Lamontagne et al., 2021). A possible opportunity, then, is to learn from studies integrating multiple -omics data to study these disease areas. We may be able to apply knowledge gained about multi-omics data integration to other disease states to elucidate disease mechanisms.

Plasma and serum were primarily used in metabolomics analyses. It is advantageous to use plasma and serum for studies investigating the metabolome because blood contains metabolites from tissues throughout the body, which enables metabolomics performed on these specimens to provide an integrative snapshot of overall metabolic status in the human body (Chetwynd et al., 2017; Turi et al., 2018). Stool was typically examined for microbiomics studies. This was an expected result as most of the current human microbiome studies have focused on the gut. Analyzing stool biospecimens allows for insight into host-microbe chemical interactions in the gut, and a snapshot of endogenously occurring metabolites from both host and gut microbiota (Turi et al., 2018). For epigenomics, genomics, and transcriptomics studies, tissue was the most common biospecimen specified. Changes in an individual’s epigenome are reflected in spatiotemporal and compartmental changes observed in their tissues (Relton & Davey Smith, 2012; Verma et al., 2014). The same is true for an individual’s transcriptomic status. Using tissue as the biospecimen for epigenomics and transcriptomics studies provides region-specific information corresponding to the sampled tissue. Tissue is a preferred biospecimen for genomics as well. Plasma, serum, and tissue biospecimens were the top biospecimens specified for proteomics studies in the portfolio. Plasma and serum are rich in circulating protein markers (e.g., alternative splicing isoforms, chemical modifications, protein cleavages, altered complexes, and altered dynamics of protein sorting and release) from many tissues in the body, which can provide researchers a global view of an individual’s proteomic state and correlated health status (Taguchi & Hanash, 2013). Additionally, since the proteome is sensitive to changes at the epigenome and transcriptome levels, proteomic state varies spatiotemporally in a tissue-specific manner as well, making tissue a favorable biospecimen choice for localized proteomics analyses. The preference for using plasma, serum, and stool in multi-omics studies may point to an objective of researchers to use less or non-invasively collected biospecimens for -omics investigations. Plasma, serum, and stool are also relatively easy to store long-term and researchers can reliably obtain these biospecimens in large quantities. Procuring tissue samples is more invasive, though necessary for conducting epigenomics and transcriptomics studies.

In the NIH grants portfolio examined, we observed that grants leverage different biospecimens for various -omics approaches. Variation in preferred biospecimen for each -omics type (e.g., choice of biospecimen for genomics studies may not be well-suited for proteomics or transcriptomics studies) is an example of multi-omics incompatibility that poses a challenge for integrated data analyses; it is not always possible or appropriate to generate multi-omics data from a single biospecimen type (Pinu et al., 2019; Worheide et al., 2021). Additionally, -omics technologies differ in their analytical platforms (Horgan & Kenny, 2011; Sun & Hu, 2016). For instance, transcriptomics is performed through microarray and RNA sequencing analytical techniques, whereas metabolomics is generally performed through nuclear magnetic resonance spectroscopy and chromatography-mass spectrometry. Using different biospecimens in combination, then, requires thoughtful experimental design. One recommendation is to plan appropriately for all -omics analyses included in a multi-omics study and prioritize study design elements such as longitudinal sample collection, biospecimens and volumes, measurement standards, analytical platforms, and other experimental considerations accordingly.

This cross-sectional evaluation of the NIH grants portfolio suggests some potential opportunities to expand the use of metabolomics data in multi-omics studies that use data integration approaches, as well as a few challenges. Integration of other -omics data with metabolomics data has potential to help researchers elucidate disease mechanisms through identifying causal determinations and functional interpretations of disease. Integration may also help improve the sensitivity of biomarkers identified via metabolomics methods. However, challenges in metabolomics research can create barriers for successful multi-omics data integration. The result that most multi-omics grants in our portfolio did not integrate metabolomics data beyond incorporating one additional -omics data type suggests that integration of increasingly more complex data is a current challenge and an impediment in the field. On July 22, 2020 the NIH released Funding Opportunity Announcements (PAR-20-276 and PAR-20-277) to support secondary and integrative analyses on existing genomics datasets for elucidating cancer outcomes, encouraging investigators to find new and innovative ways to use previously collected data (National Institutes of Health, 2020a, 2020b). In a similar vein, perhaps some attention can be directed toward understanding how already established -omics datasets are being leveraged and integrated together. Data compatibility is also an issue as -omics data are widely variable and -omics approaches vary in their methods, biospecimen requirements, validated standard availability, and analytic techniques. Our analysis presents a future opportunity for greater investment of training and career development awards in multi-omics research.

Limitations to this study include that we only examined data from NIH grants and these data may not be a direct reflection of what is currently published in the literature. However, these data show various applications of multi-omics approaches to study human health and disease in the examined NIH grants portfolio, funded by one federal agency in the United States. These data are also useful for understanding the support for integration of metabolomics data as a component of multi-omics studies, while also highlighting areas of potential opportunity that may benefit from further investment to confront or overcome challenges present in the field.

NIH-funded multi-omics studies that include metabolomics performed integration to a limited extent by primarily incorporating only one other -omics data type. A number of challenges need to be addressed for metabolomics data to be more widely analyzed in multi-omics studies, including increasing confidence in metabolite identification, addressing -omics data incompatibility, overcoming resource requirements for integrating multiple -omics data, selecting appropriate study designs that support multi-omics research, and addressing variability between -omics approach requirements.

## Supplementary Information

Below is the link to the electronic supplementary material.

Supplementary file1 (DOCX 18 kb)

Supplementary file2 (DOCX 56 kb)

Online Resource 3 Data used to conduct the NIH-funded grants portfolio analysis (PNG 43 kb)

Supplementary file4 (XLSX 31 kb)

## Data Availability

The data used to conduct this portfolio analysis can be found in the supplementary material, Online Resource 3.

## Declarations

### Disclaimer

The data in this publication have only been vetted by the authors, and no additional experts at other NIH Institutes and Centers have reviewed the content presented. These data do not represent official NIH data, and this analysis was not performed to inform the NIH on this scientific area. Rather, the portfolio analysis was used for the purpose of informing the authors from the National Cancer Institute, the National Institute of Diabetes and Digestive and Kidney Diseases, and the NIH Office of Nutrition Research.
