# Supplementary material for: An evaluation of the National Institutes of Health grants portfolio: identifying opportunities and challenges for multi-omics research that leverage metabolomics data
Source: Metabolomics. 2022 Apr 30;18(5):29. doi: 10.1007/s11306-022-01878-8 (PMC9056487; doi:10.1007/s11306-022-01878-8)
Supplement: Supplementary file 1 — Supplementary file1 (DOCX 18 kb) [file 11306_2022_1878_MOESM1_ESM.docx]

**Journal name: *Metabolomics***

**Title:** An Evaluation of the National Institutes of Health Grants Portfolio: Identifying Opportunities and Challenges for Multi-Omics Research that Leverage Metabolomics Data

**Authors:** Catherine T. Yu, Brittany Chao, Rolando Barajas, Majda Haznadar, Padma Maruvada, Holly L. Nicastro, Sharon A. Ross, Mukesh Verma, Scott Rogers, Krista A. Zanetti

**Corresponding Author:**

Krista A. Zanetti, PhD, MPH, RD

Affiliation: Division of Cancer Control and Population Sciences, National Cancer Institute, Rockville, MD, USA

Email: zanettik@mail.nih.gov

| **Online Resource 1.^[[1]](#footnote-1)^** Types of grant programs represented in the multi-omics portfolio | |
| --- | --- |
| Research Grants (R Series) | Research Grants are awarded to fund biomedical research conducted by various types of professional institutions and independent researchers. |
| Career Development Awards (K series) | Career Development Awards support senior post-doctoral or faculty-level scientists to support independent research and competitiveness for future funding opportunities. |
| Cooperative Agreements (U Series) | Cooperative Agreements are awarded to support projects in high-priority research areas demanding sizable NIH staff engagement that is beyond what is generally  required for R or P Series grants. |
| Program Project/Center Grants (P series) | Program Project/Center Grants support extensive research efforts that involve multiple projects. |
| Institutional Training and Director Program Project Awards (D Series) | Institutional Training and Director Program Project Awards support grants to both new and established researchers, who have the potential to make extraordinary contributions to biomedical research. |
| Fellowship Grants (F Series) | Fellowship Grants support students and scientists across the career continuum to advance their experience conducting research. |
| Non-HHS Research Projects (I Series) | I Series grants are awarded to fund non-DHHS entities. |

**Online Resource 1** Table describing NIH grant mechanisms represented in the multi-omics portfolio

1. <https://grants.nih.gov/grants/funding/funding_program.htm> [↑](#footnote-ref-1)
