# Supplementary material for: An evaluation of the National Institutes of Health grants portfolio: identifying opportunities and challenges for multi-omics research that leverage metabolomics data
Source: Metabolomics. 2022 Apr 30;18(5):29. doi: 10.1007/s11306-022-01878-8 (PMC9056487; doi:10.1007/s11306-022-01878-8)
Supplement: Supplementary file 2 — Supplementary file2 (DOCX 56 kb) [file 11306_2022_1878_MOESM2_ESM.docx]

**Journal name: *Metabolomics***

**Title:** An Evaluation of the National Institutes of Health Grants Portfolio: Identifying Opportunities and Challenges for Multi-Omics Research that Leverage Metabolomics Data

**Authors:** Catherine T. Yu, Brittany Chao, Rolando Barajas, Majda Haznadar, Padma Maruvada, Holly L. Nicastro, Sharon A. Ross, Mukesh Verma, Scott Rogers, Krista A. Zanetti

**Corresponding Author:**

Krista A. Zanetti, PhD, MPH, RD

Affiliation: Division of Cancer Control and Population Sciences, National Cancer Institute, Rockville, MD, USA

Email: [zanettik@mail.nih.gov](mailto:zanettik@mail.nih.gov)


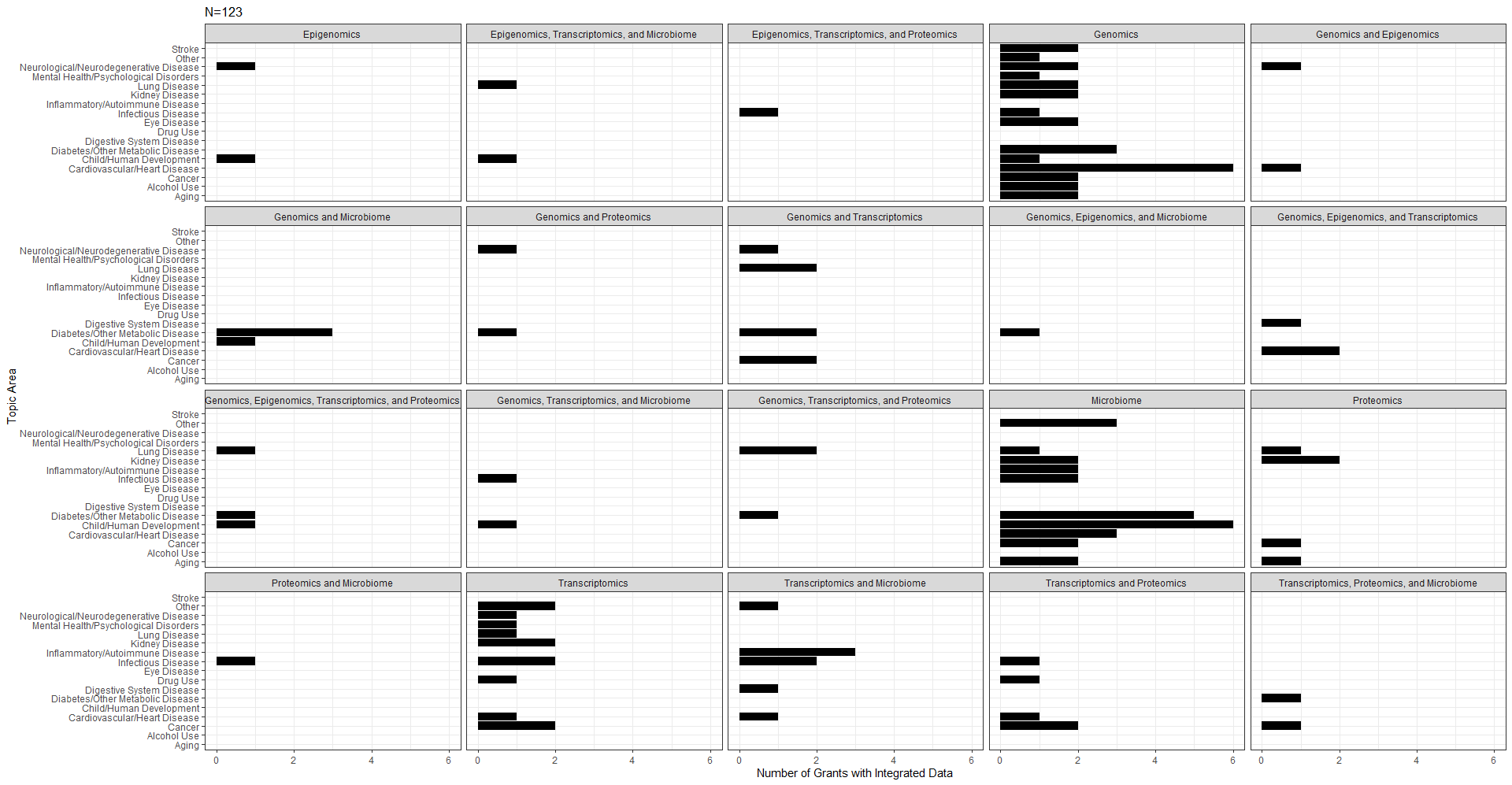


**Online Resource 2** Bar graphs depict the number of NIH-supported grants employing metabolomics in multi-omics studies with integration with one or more other -omics data by topic area studied, facetted by various -omics combinations integrating data with metabolomics data
