# Supplementary figures and images for: An evaluation of the National Institutes of Health grants portfolio: identifying opportunities and challenges for multi-omics research that leverage metabolomics data
Source: Metabolomics. 2022 Apr 30;18(5):29. doi: 10.1007/s11306-022-01878-8 (PMC9056487; doi:10.1007/s11306-022-01878-8)

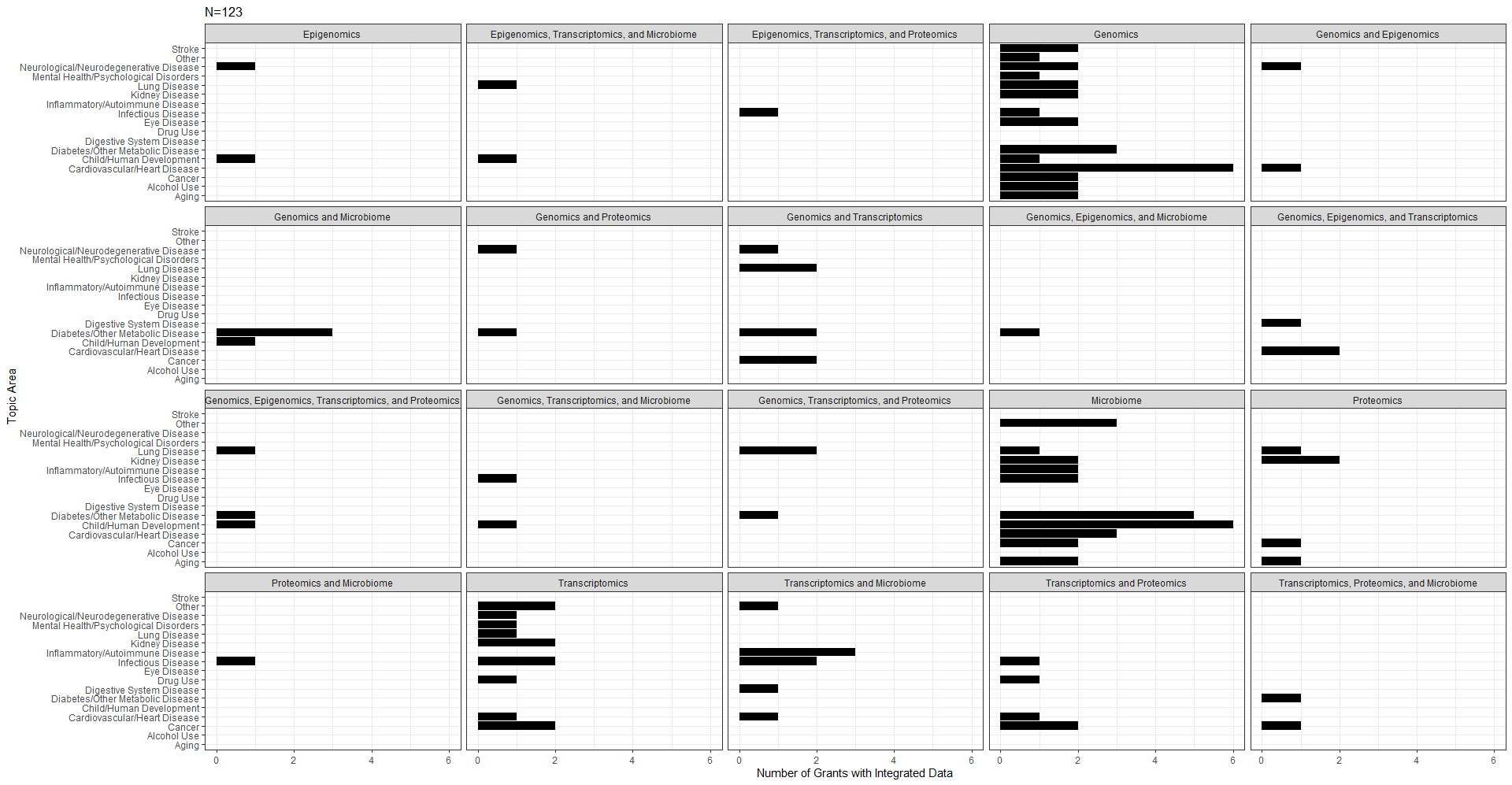

Supplement: Supplementary file 3 — Online Resource 3 Data used to conduct the NIH-funded grants portfolio analysis (PNG 43 kb) [file 11306_2022_1878_MOESM3_ESM.png]
